# Influence of Changes in the Shape of the Anode Channel in Polymer Electrolyte Fuel Cell on the Loss of Its Service Life

**DOI:** 10.3390/ma14237338

**Published:** 2021-11-30

**Authors:** Daniel Sławiński, Michał Soszko, Wojciech Tokarz, Sebastian Bykuć

**Affiliations:** 1 Institute of Fluid-Flow Machinery Polish Academy of Sciences, Fiszera 14 Str., 80-321 Gdańsk, Poland; sebastian.bykuc@imp.gda.pl; 2 Łukasiewicz—Industrial Chemistry Institute, Rydygiera 8 Str., 01-793 Warsaw, Poland; michal.soszko@ichp.pl (M.S.); wojciech.tokarz@ichp.pl (W.T.)

**Keywords:** graphite, rupture life, low-cycle fatigue

## Abstract

The fuel cell operation is associated with significant current density and durability problems, among other anode collectors. We used a numerical model based on flows with chemical reactions in a porous medium to solve these problems. We tested four variants of the anode channels. In the shape of the anode channel, we introduced changes to improve the current density. We also examined the influence of the channel shape on the stress field and rheological processes in the casing material. We verified the numerical model on the experimental data. Furthermore, we corrected the amount of the hydrogen stream and the produced water in the whole range of the cell’s operation. The test results show that it is possible to increase the current density in all operating fields of the fuel cell while maintaining a low mechanical load on graphite elements and their safe operation time.

## 1. Introduction

The PEMFC hydrogen cells have their origins in the 1970s in works for space flights. Nowadays, it seems that this solution may be significant, which could potentially alter the current approach to the energy sector by becoming an alternative to burning conventional fossil fuels. The great advantage of this technology is the lack of CO_2_ generation downstream in the process and no harmful combustion products such as NO_X,_ etc. The use of PEMFC cells is associated with the development of hydrogen technology, and its niches may be found in stationary (small energy) and mobile applications. 

The PEMFC (Polymer Electrolyte Fuel Cell) is comprised of a negatively charged electrode (anode) and a positively charged cathode separated by a polymer electrolyte in the form of a membrane. The advantage of this solution is high current density, low operating temperature, short start-up time, low weight, and dimensions. Hydrogen is fed through the anode and oxygen is fed to the cathode. The membrane is permeated, from the anode to the cathode, solely by the protons, while the electrons flow from the anode to the cathode in the external circuit, bypassing the membrane. The input substrates, as already mentioned, are 2H_2_ (hydrogen) and O_2_ (oxygen), and the end product is 2H_2_O (water). 

Technology, despite developing for many years, has been struggling with specific problems. One of the key features is the low current density plus the durability of the polymer membrane and body of the channel. There are several ways to improve the power density. One of the commonly used is the aerodynamic optimization of the channels [1,2]. Properly selected fuel cell operating parameters and reduction of flow resistance allow for improving the density power generation from 4.7% to 7%. 

The temperature gradient in the cathode gas diffusion (GDL) layer greatly influences the improvement of the current density of the PEMFC cell. On the other hand, the too low temperature gradient may cause problems with the fuel cell start-up and the membrane’s durability [3,4]. The stabilization of the temperature made it possible to distribute the reactants faster, control the water transport, and, thus, restore the performance in the initial time of the fuel cell’s operation. 

The third trend contributing to the improvement of the current density in the fuel cell is the drainage of water from the channels. To improve water transport, partitions that slowed down the flow were introduced into the geometry of the channels [5,6]. Separators made as rigid plates or channel narrowings collect the water that is formed, preventing the flooding of significant areas of active exchange. Thanks to these measures, the fuel cell’s power increased by 21% compared to the reference cell with constant dimension channels in a serpentine arrangement (CSFF). There is no data in the literature on the influence of these separators on the durability of the channels and polymer membrane. 

The use of open flow channels [7,8,9,10,11] stabilizes the operation of the fuel cell, with a significant increase in flow resistance. However, an excessive increase in the pressure difference affects the membrane’s durability, deformation of the channels, and the tightness of the cell’s operation. Higher pressures mean significant deformations of the bodies and, thus, trouble in constructing stacks of fuel cells caused by the excessive strain of the materials used. The mentioned aspects have a significant influence on the number of safe loads of the fuel cell. 

Our research niche was to improve fuel cell current density by introducing wave sinusoidal fluid flow (WSFF). The appropriately selected sinusoidal shape [12,13,14] influenced the flow similar to the discussed open channel flow. Reducing the cross-sectional area forced a local increase in the flow velocity of the medium and, thus, an increase in the mass flow flowing through the membrane [15,16,17,18]. 

The advantage of WSFF is working at much lower flow pressures, which directly affects the strains and durability of the materials. The obtained results were related to the current–voltage characteristics of the CSFF serpentine cell. We have examined the distribution of strains and stresses for each considered variant of the anode channel. Subsequently, we examined the influence of shape changes on low-cycle fatigue and durability on high-temperature creep.

## 2. Materials and Methods

### 2.1. Problem Formulation 

**Figure 1:**
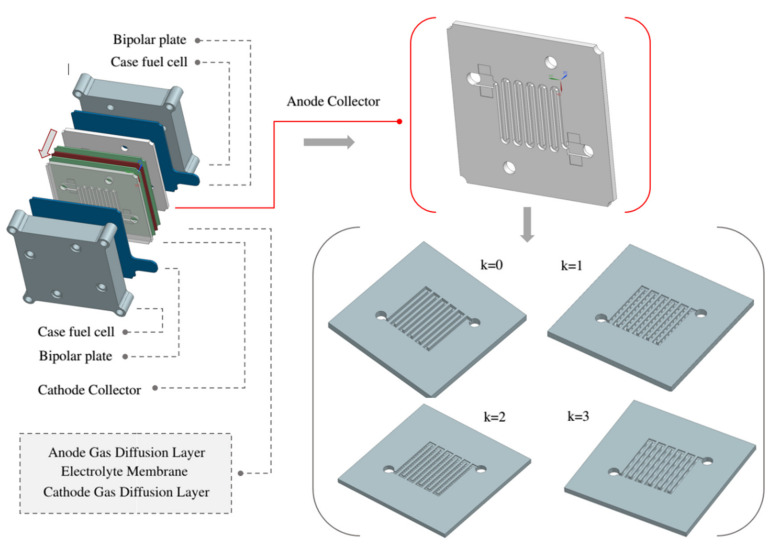
Physical model of fuel cells with proton exchange membrane.

The analyzed cell model consisted of two electrodes, an anode and cathode, separated by a polymer electrolyte layer. Both electrodes had serpentine channels, symmetrically positioned in relation to each other. Hydrogen was fed to the anode channels, while air flowed in the cathode channel. The channels were separated from each other by a polymer membrane (MEA), as presented in Figure 1. The polymer membrane used for the transport of hydrogen protons consisted of successive layers of an anode gas diffusion layer (electrode) and a symmetrical GDL cathode [19]. Both layers operated as electrodes discharging the electric charge by means of an external circuit. Then, the catalytic layers on both sides, where chemical reactions took place, were, successively:

(1) $$H_{2}⇐\Rightarrow 2H^{+}+2e^{-}\;\;(\mathrm{anode}\;\mathrm{TPB})\;$$

(2) $$\frac{1}{2}O_{2}+2e^{-}+2H^{+}⇐\Rightarrow H_{2}O\;\;(\mathrm{cathode}\;\mathrm{TPB})$$

The electrons produced in the anode traveled through the outer circuit to the cathode, while protons (H^+^) traveled through the membrane from the TPB anode layer to the cathode layer (TPB). 

Successive portions of water were produced at the cathode following osmotic pressure and electrochemical reactions. Water vapor, above the saturation pressure, turned into a liquid state. The production of water vapor and its subsequent transformation into a liquid state is a prerequisite for the correct and continuous operation of a PEMFC fuel cell. 

We have examined variants of the anode channel shape in the study to improve the current density in the fuel cell. The form of the CSFF channel in the k = 0 variant coincided with the experimental anode channel, which allowed for the correct validation of the numerical model with the measurement data.

After verifying the model, we changed the shapes of the anode channel by inserting successive WSFF variants shown in Figure 1. Then, with the same boundary conditions and mesh sizes, we repeated the numerical analysis. Finally, we compared the results obtained from the WSFF channels with the reference results from the CSFF channel during the discussions.

Additionally, we investigated the influence of changes in the channel shape on the strain field and stresses in the anode collector casing. Finally, after comparing the results, we analyzed their effect on the rheological processes in the material. Namely, creep-rupture and low-cycle fatigue were analyzed using the regression analysis technique and Coffin–Manson procedure, respectively.

The next sub-section gives describes and discusses the detailed dimensions of the analyzed variants of the anode channel. The material properties and operating conditions of the modeled fuel cell [20,21] are presented in Table 1 and Table 2. 

#### Variants of the Modified Shapes of the Anode Channels

**Figure 2:**
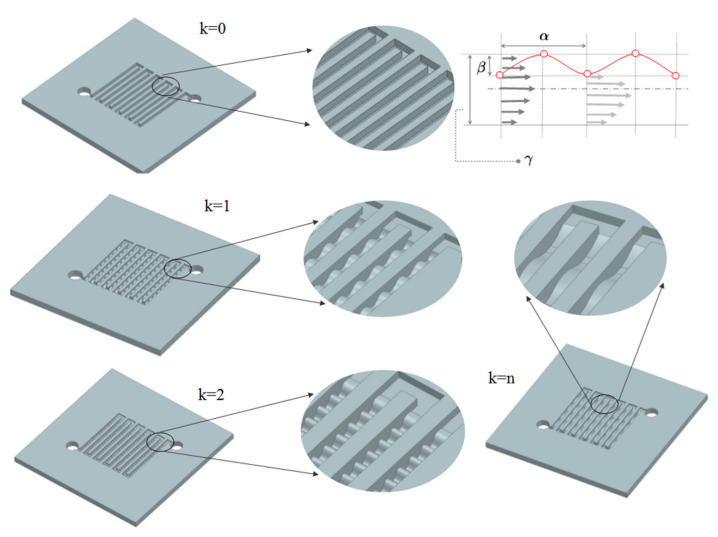
Modified shapes of the anode channel design in a PEMFC fuel cell.

Figure 2 shows considered variants of the anode channel shape. The first variant, k = 0, is a constant-height channel in a serpentine pattern, and the others have sinusoidal ripples on the upper surface. The wave depth in each case reached 0.5 of the channel height, and the changing parameter was the distance between the wave tops. In the k = 2 variant, the distance between the wave peaks was smaller than the channel height, while in the k = 1 and k = 3 variants, this distance has been equal and greater, respectively. Detailed dimensions are given in Table 3. 

### 2.2. Mathematical Formulation

#### 2.2.1. Conservation Equations

The mathematical model is based on the following assumptions [22,23]:

- The cell operates under steady-state conditions;
- The reacted gases are incompressible ideal gases;
- The fluid flow is laminar because the Reynolds number is less than 2000;
- MEA is an isotropic porous media;
- The cell operates at a steady-state temperature.

#### 2.2.2. Conservation of Mass 

For multi-component flows with chemical reactions for the channel area and the porous medium, the mass conservation equation was recorded as follows [24]:

(3) $$\frac{\partial }{\partial t}\left(Y_{i}\rho_{i}\right)+\mathrm{div}\left(Y_{i}\rho_{i}v\right)=S_{i}$$

(4) $$\frac{\partial }{\partial t}\left(Y_{i}\rho_{i}\right)+\mathrm{div}\left(Y_{i}\rho_{i}v\right)=\mathrm{div}\left(J_{i}\right)+S_{i}$$

where $\rho_{i},\;v,\;S_{i},$ is, sequentially, the density of the respective component, flow velocity, and mass source defined by a separate equation, resulting from the presence of chemical reactions. 

An additional element, $J_{i}$ describing the diffusion flux in the considered layer appears here for the porous medium, and $Y_{i}$ is the size of the mass fraction, $Y_{i}\equiv \rho_{i}/\rho$. 

The source term, $S_{i}$, describing the mass degradation of the i-th reagent and the increase on the side of the reaction product is defined by the equations:

(5) $$S_{H_{2}}=-\frac{M_{w,H_{2}}}{2F}\;R_{an}<0\;$$

(6) $$S_{O_{2}}=-\frac{M_{w,O_{2}}}{2F}\;R_{cat}<0$$

(7) $$S_{H_{2}O}=-\frac{M_{w,H_{2}O}}{2F}\;R_{cat}<0$$

(8) $$R_{an}=\left(\zeta_{an}j_{an}^{ref}\right){\left(\frac{A}{A_{ref}}\right)}^{\gamma_{an}}\left(e^{\alpha F\eta_{an}/RT}\right)$$

(9) $$R_{cat}=\left(\zeta_{cat}j_{cat}^{ref}\right){\left(\frac{A}{A_{ref}}\right)}^{\gamma_{cat}}\left(e^{\alpha F\eta_{cat}/RT}\right)$$

where *M_w’i_* is equivalent weight, *R_an,cat_* is a source term described by the Tafel formula, $j^{ref}$ reference exchange current density per active surface area, ζ is specific active surface area, A, A_ref_ is local species concentration and reference value, γ is concentration dependence, α is transfer coefficient, and *F* is the Faraday constant 9.65 × 10^7^. 

#### 2.2.3. Conservation of Electric Charge

When we assume that the electric current is carrying charged charges, the mass balance equation will also apply to this case [25]:

(10) $$\frac{\partial }{\partial t}\left(e_{i}\rho_{i}\right)+\mathrm{div}\left(e_{i}\rho_{i}\;v\right)=\mathrm{div}\left(e_{i}J_{i}\right)+S_{e}$$

where $e_{i},\;S_{e}$ are, successively, the electric charge and the source element.

Since the amount of electric charge does not change in the course of electrochemical reactions, the following also remains valid:

(11) $$\overset{n}{\underset{i=1}{\sum }}S_{e}=0$$

Therefore, we obtained the local balance equation for the electric charge density $\left(e_{i}\rho_{i}\right)$ of the respective component. $e_{i}J_{i}$ describes the component’s contribution to the diffusive current density, while element $e_{i}\rho_{i}\;v$ describes the component’s contribution to the convective current density. 

By adding up all components of the fluid and by introducing the dependence $e=\sum_{i}e_{i}\rho_{i}\;,$ the Equation (10) adopts the following form:

(12) $$\frac{\partial \;}{\partial t}e=-\mathrm{div}\left(I+ev\right)$$

where e is the spatial charge, while

(13) $$I=\sum_{i}e_{i}J_{i}$$

is the total diffusive current density and ev describes the total convective electric current density generated in the PEMFC fuel cell [26]. 

#### 2.2.4. Electrochemical Equations Describing the Three Types of Losses 

The formula shows the value of activation losses:

(14) $$\Delta V_{act}=\frac{RT}{\alpha \;n\;F}\;\log\frac{i}{i_{0}}$$

where α is the electron transfer coefficient of the reaction at the electrode and *i_0_* is the exchange current density. 

Because the electrolyte and fuel cell electrodes obey Ohm’s law, the ohmic losses were written by the equation:

(15) $$\Delta V_{ohm}=i\;R_{ohm}$$

where *i* is the current flowing through the cell and *R_ohm_* is the total cell resistance, which includes electronic, ionic, and contact resistance $R_{ohm}=R_{electronic}+R_{ionic}+R_{contact}$. 

The value of losses due to the change of concentration at the electrode is described by the formula:

(16) $$\Delta V_{con}=\frac{RT}{n\;F}\;\ln\left(1-\frac{i}{i_{Limit}}\right)$$

Summing up the individual elements additively, the total amount of losses was described by the formula:

(17) $$V=V_{R}-\Delta V_{act}-\Delta V_{ohm}-\Delta V_{con}$$

where *V_R_* is the theoretical value of electric potential in fuel cell, *V_R_* = 1.17 V. 

#### 2.2.5. A Low-Cycle Fatigue Model Based on Coffin–Manson’s Criteria 

The leading causes of thermal fatigue are mechanical deformations resulting from blocking the movement of connections. These connections prevent the free movement of machine parts as the result of cyclical temperature changes. Thermal fatigue is considered a process in a limited number of cycles. This process is characterized by crack formation mechanisms similar to creep and mechanical fatigue. Therefore, it is treated as an accumulation of two processes: cyclic deformation (primarily plastic) and creep. The criterion given by Coffin–Manson proves correct for the cases of thermal fatigue [27]:

(18) $$\Delta \varepsilon =\varepsilon_{f}^{0.6}N_{f}^{-0.6}+3.5\frac{Rm}{E}\;N_{f}^{-0.12}\;$$

where $\varepsilon_{f}$ corresponds to deformations during a static tensile test, $N_{f}$ is the number of cycles required to destroy the material, and Rm, E are, respectively, the tensile strength during the static test and Young’s modulus. Material data depends on temperature. 

The small strain tensor was written as Green’s tensor linearization and expressed by symmetric combinations of displacement gradients u:

(19) $$\varepsilon =\frac{1}{2}\;\left(\mathrm{grad}\;u+{\mathrm{grad}}^{T}\;u\right)$$

The strain tensor deviator is written us as:

(20) $$\varepsilon^{d}=\varepsilon -\frac{1}{3}\;\mathrm{tr}\;\left(\varepsilon \right)I$$

where I is the Gibbs identity tensor $I=\delta_{ij}e_{i}⊗e_{j}.$

The scalar of the strain tensor is written as the second minor invariant of $J_{2s}$ from the strain deviator:

(21) $$J_{2s}=\frac{1}{2}\varepsilon_{ij}^{d}\varepsilon_{ji}^{d}=\frac{1}{2}\;\varepsilon^{d}\cdot \varepsilon^{d}$$

(22) $$\varepsilon_{eq}=\sqrt{-J_{2s}}=\sqrt{\frac{2}{3}\;\varepsilon^{d}\cdot \varepsilon^{d}\;}\;$$

#### 2.2.6. A Creep Rupture Model Based on Regression Analysis Technique 

To estimate the time required for the destruction of material due to creep as a function of stress and temperature, we used formulas developed on experimental results at the Oak Ridge National Laboratory (ORNL), Idaho National Engineering Laboratory, and General Electric (GE) consortium:

(23) $$\log t_{r}=C_{h}-193.662\;\log\sigma +88.117{\left(\log\sigma \right)}^{2}-12.807\;{\left(\log\sigma \right)}^{3}-0.01052\;T\log\sigma$$

where the base for all logarithms is 10, and $t_{r}$ is the rupture life (h), σ is stress (MPa), and *T* is the temperature (K). 

The stress tensor deviator is written us as:

(24) $$\sigma^{d}=\sigma -\frac{1}{3}\;\mathrm{tr}\;\left(\sigma \right)I$$

However, we presented the reduced stresses as a combination of the first and second main invariants $I_{\sigma },\;II_{\sigma }$ written in the form:

(25) $$I_{\sigma }=\mathrm{tr}\;\sigma =\sigma_{ii}$$

(26) $$II_{\sigma }=\frac{1}{2}\;\mathrm{tr}\;\sigma^{2}+\mathrm{tr}\;\left(\sigma^{2}\right)=\frac{1}{2}\left(\sigma_{ii}\sigma_{ij}-\sigma_{ij}\sigma_{ij}\right)$$

(27) $$\sigma_{eq}=\sqrt{I_{\sigma }^{2}-3II_{\sigma }}=\sqrt{\frac{3}{2}}\;\sqrt{\sigma_{ij}\sigma_{ij}-\frac{1}{3}\sigma_{ii}\sigma_{jj}}$$

After introducing the definition of the second minor invariant $J_{2s}$ from the stress deviator, the stress scalar takes the following form:

(28) $$J_{2s}=II_{\sigma }+\frac{1}{3}\;I_{\sigma }^{2}=\frac{1}{2}\left(\sigma_{ij}^{d}\;\sigma_{ij}^{d}\right)$$

(29) $$\sigma_{eq}=\sqrt{-J_{2s}}=\sqrt{\frac{3}{2}\;\sigma^{d}\cdot \sigma^{d}}$$

### 2.3. Validation of the Numerical Model 

**Figure 3:**
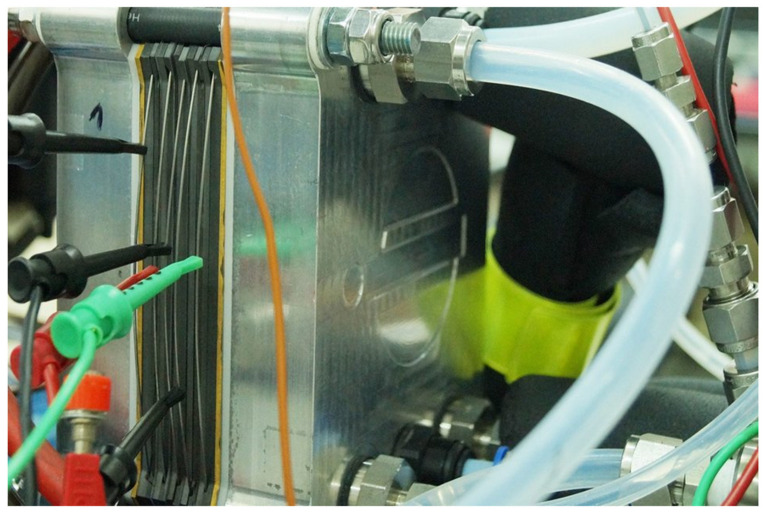
The experimental model of a PEMFC fuel cell equipped with a CSFF anode channel.

We validated the numerical model in cooperation with Łukasiewicz, Industrial Chemistry Research Institute in Warsaw. The CSFF fuel cells with dimensions identical to the numerical variant k = 0 were used for validation, Figure 3. We compared the results of both fuel cells across the entire range of work. 

**Figure 4:**
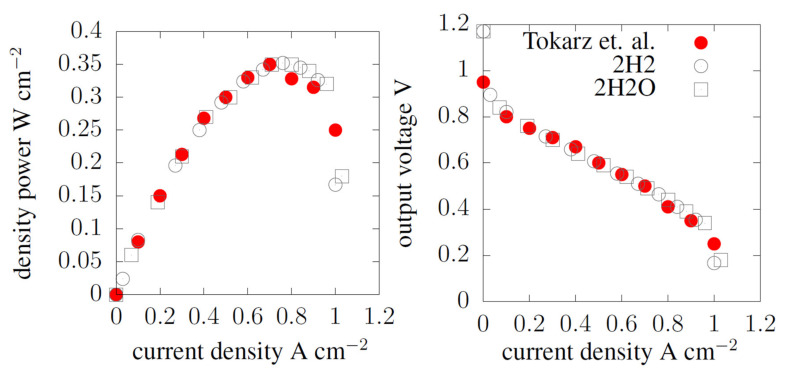
The polarization curve of the current–voltage and power density characteristic for experimental and numerical data [28]. Reproduced with permission from W. Tokarz, P. Piela, Int J Hydrogen Energy; published by Elsevier, 2016.

We used the commercial research code Ansys Fluent to perform numerical simulations of computational fluid dynamics. We made the finite element mesh for Computational Solid Dynamics simulations and the finite volumes mesh for Computational Fluid Dynamics simulations in the commercial ICEM Ansys code. A multi-component flow model with chemical reactions in a porous medium was used. The results of both characteristics, experimental and numerical, are shown in Figure 4. The work parameters used to validate the numerical model with the experimental data are shown in Table 4.

For greater precision, the numerical model was verified for two streams read in the cross-section of the cathode catalyst layer (TPB). The amount of hydrogen *J_i_ =* 2H_2_ and water *J_i_ =* 2H_2_O produced during the reaction is described in Equation (2). After inserting into Equation (8), both sizes of streams showed good convergence with the measurement data, as shown in the discussed graph. 

## 3. Results and Discussion

### 3.1. Comparison of Current–Voltage Characteristics for the CSFF Fuel Cell and the WSFF Variants of Fuel Cells 

**Figure 5:**
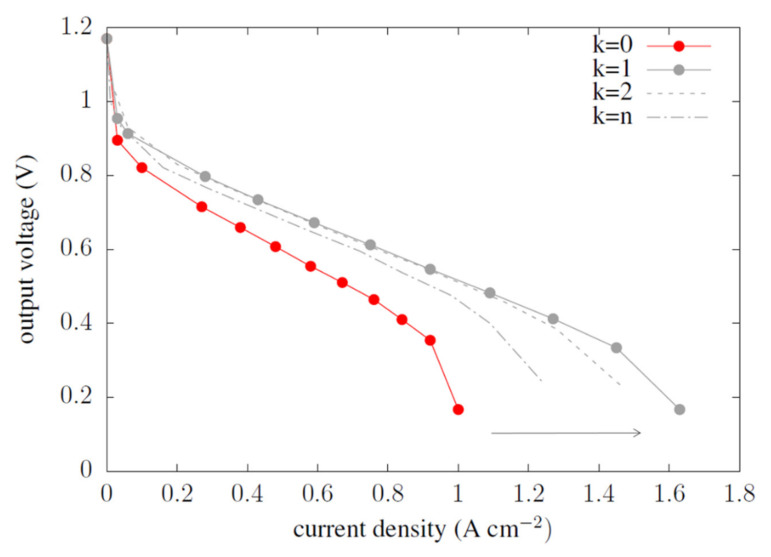
Comparison of the current–voltage characteristics for the reference fuel cell and following the optimization of the anode channel.

In the figure below (Figure 5), the comparison of the current–voltage characteristics obtained for WSFF fuel cells in many types with fuel cell CSFF in type k = 0 is presented. Each modification of the shape with the use of identical operating conditions of the cell yielded an improvement in the cell characteristics. Most optimal was the combination of k = 1. The maximum current flux density obtained was 1.6 A cm^−2^, with the reference value equal to 1 A cm^−2^. Both combinations with wide k = n, and narrow k = 2 corrugations yielded lower results than k = 1.

**Figure 6:**
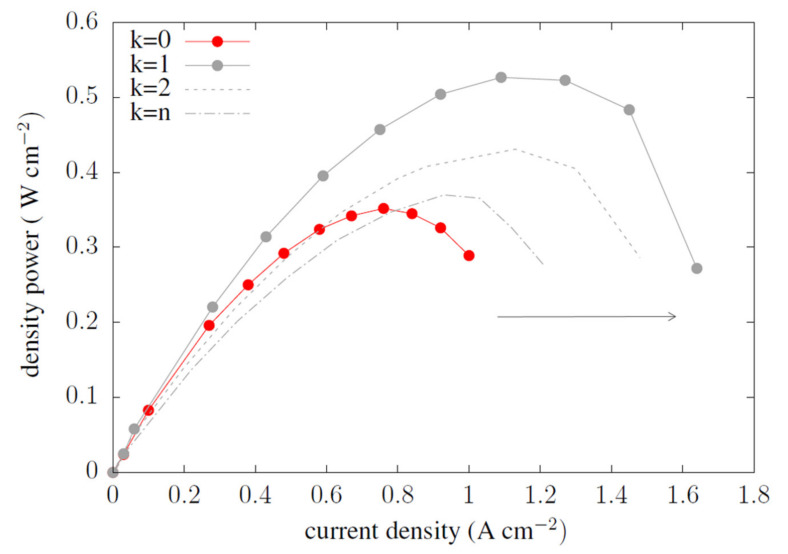
Graph of the power density generated in the reference fuel cell and after optimization of the anode channel.

The graph in Figure 6 presents the shift of the cell’s maximum power density area to the upper areas of the graph. For example, from the maximum range 0.3 W cm^−2^ of the reference cell k = 0, the optimal shape generated power of the order 0.5 W cm^−2^, with current densities ranging from 1.1 to 1.22 W cm^−2^. The k = n compared from the CSFF fuel cell in the variant k = 0 only slightly improved the current density. 

### 3.2. A field of Concentration of Individual Components in CSFF and WSFF Fuel Cell 

**Figure 7:**
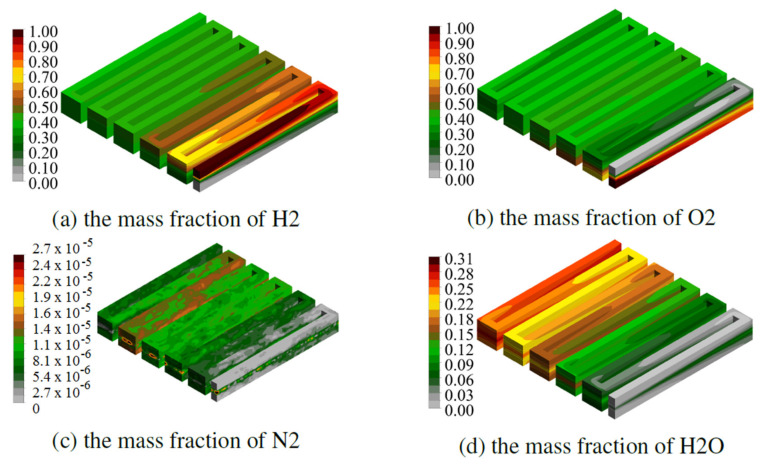
Field of mass fractions of components, obtained for half the power of the reference fuel cell.

**Figure 8:**
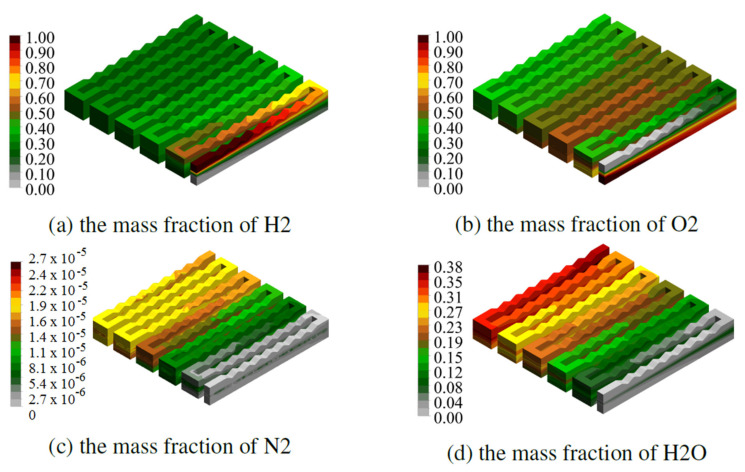
The field of mass fraction of components, obtained for half power of the WSFF fuel cell in the variant k = 1.

Figure 7 and Figure 8 show the mass fractions of individual components, read with the same operating parameters, for the fuel cell CSFF and WSFF in the variant k = 1, successively.

The high mass fractions of H_2_ for the CSFF fuel cell reach half of the cell, and the highest concentrations are along the first two channels. The geometry of the anode channel in the WSFF fuel cell in version k = 1, thanks to shape change, allows for better transport of H_2_ through the membrane. 

Due to the identity of the shape of the cathode channel in the CSFF and WSFF fuel cells, the O_2_ mass fraction fields occupy similar areas of the cell. 

The WSFF fuel cell in variant k = 1 produces more water compared to CSFF. This is due to a better-shaped anode channel and, thus, a better distribution of H_2_ inside the membrane. The greater the efficiency of electrochemical reactions, the greater the current density obtained in the fuel cell, which is presented in the figures in the next section. 

### 3.3. A Field of Current Density for the CSFF and the WSFF Variants of Fuel Cells 

This section discusses the current density field obtained for two characteristic variants; k = 0 having CSFF channels and k = 1 having WSFF channels. The shape of the k = 1 variant allowed us to obtain the most efficient I–V polarization curve. For both variants, we read the local current density map in time steps describing the operating range of the fuel cell. 

**Figure 9:**
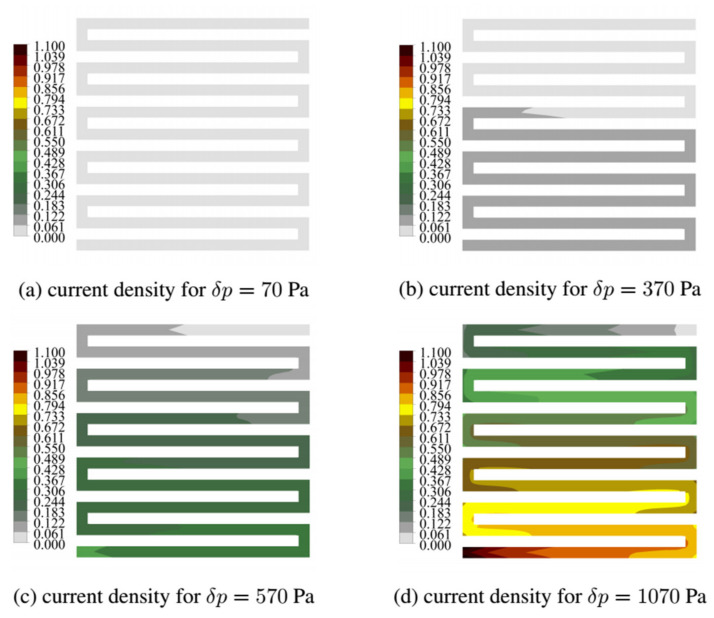
The current field generated in the cathode catalyst layer at different loads for fuel cells in variant k = 0.

Figure 9 shows the current density maps for the variant k = 0. For the initial operating range of the cell, the current read density is as low as 0.12 A/cm^2^. However, at maximum load, the current density reached the value of 1 A/cm^2^. In every load variant at the end of the fuel cell, we located the largest size of current density. 

**Figure 10:**
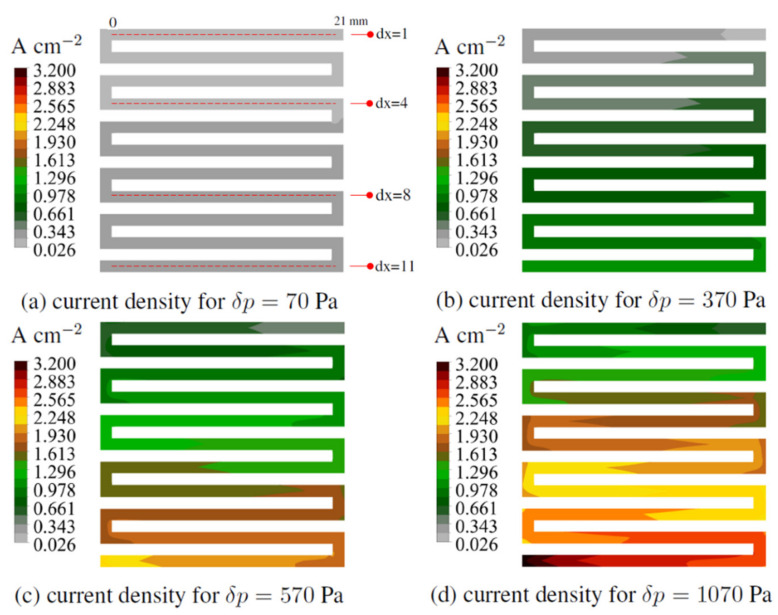
The electric current field generated in the cathode catalyst layer at different loads for fuel cells in variant k = 1.

In Figure 10, the current density for the WSFF fuel cell in the variant k = 1 is presented at different loads. The maximum current density corresponds to a pressure difference between the inlet and outlet of 1070 Pa, which amounted to 3.2 A/cm^2^. 

During the maximum load of the fuel cell, the value of the read current density was between 0.98 A cm^−2^ at the inlet and the value of 2.8 A/cm^2^ read at the outlet. Thus, the highest current density in every load variant was located at the end of the fuel cell. 

In Figure 10a, the sections along which the electric current values were read, shown in the next figure, are marked. The first segment marked as dx = 1 corresponded to the first channel. The second marked dx = 4, corresponded to the fourth channel. The third, labeled dx = 8, corresponded to the eighth, while the fourth, marked dx = 11, was the eleventh and last channel. 

**Figure 11:**
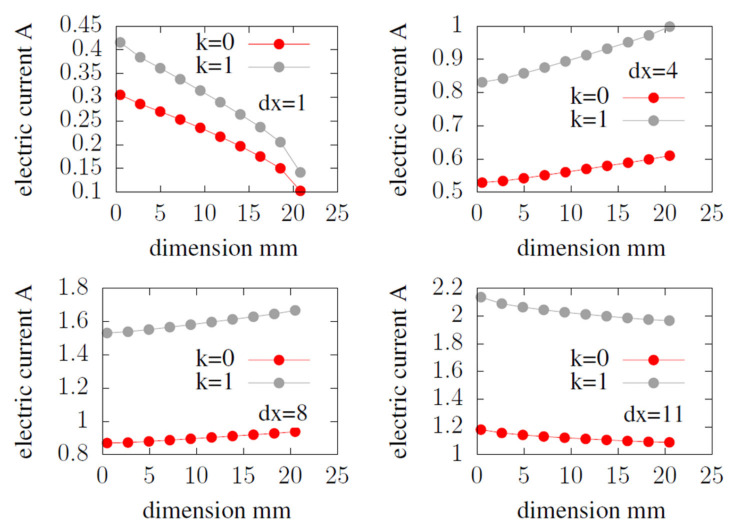
The value of electric current, read along channel length at the cathode catalyst layer.

In the Figure 11, the results read along the marked lines were compared for the WSFF fuel cell in the k = 1 variant with CSFF in the k = 0 variant. 

The WSFF fuel cell in the variant k = 1 generates a higher electric current along the considered channels’ length. The least observable difference is at the beginning of the first channel; dx = 1. With each subsequent one, the difference in the value of the electric current obtained increases. 

### 3.4. Field of Equivalent Strain and Estimated Number of Cycles to Fatigue Based on Coffin–Manson’s Criteria 

This section discusses the strain fields obtained in all variants of the anode channel. The simulations were performed with the same load and assumed marginal conditions. To illustrate the strain fields in the entire operating range of the fuel cell, the variant with CSFF channels, marked k = 0, and the variant k = 1, with the highest power density, were selected. 

**Figure 12:**
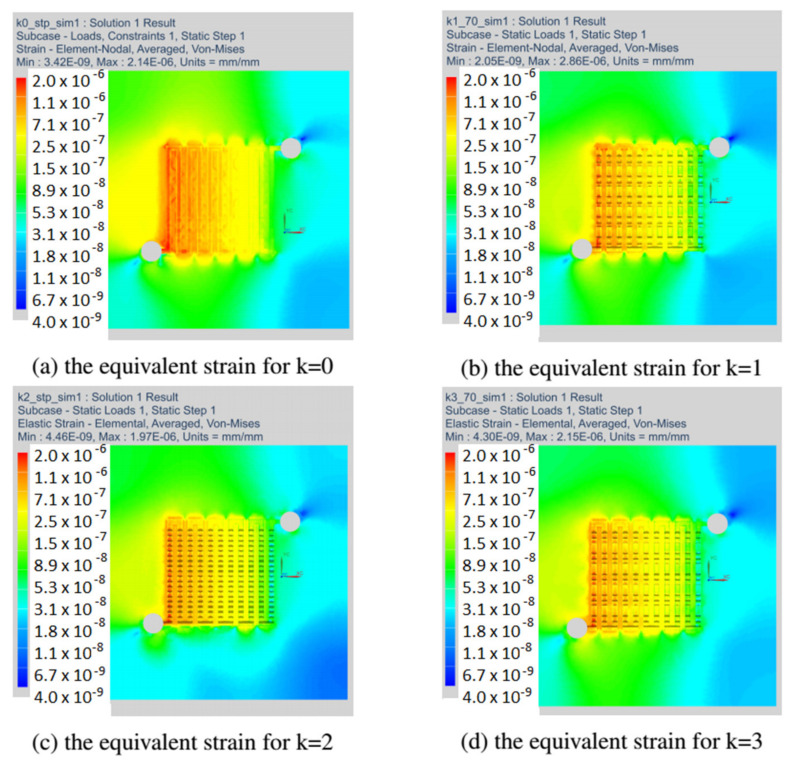
The field of equivalent strain read at a load of 70 Pa for all variants of the anode channel.

Figure 12 shows the fields of equivalent strain for all considered variants of the fuel cell. As already mentioned, the simulations were made with the identical operating parameters of the fuel cell. For all the changes in the shape of the anode channel, the strain maps do not differ significantly, and the places with the highest material strain include the first inlet channels. 

**Figure 13:**
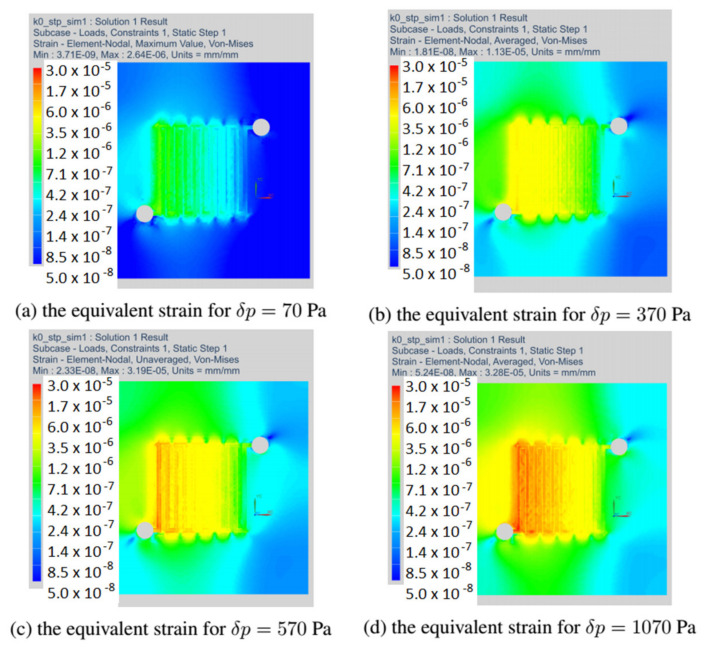
The field of equivalent strain read in the anode layer at different loads for fuel cell in variant k = 0.

In the next drawing, Figure 13, the distribution of equivalent strain fields for the variant k = 0 read at selected operating points of the fuel cell is shown. For each variant, we have located the fields of maximum strain at the beginning of the channel. The presented variant k = 0 corresponded to the CSFF channel and has the lowest current density among the considered variants of the cell. 

**Figure 14:**
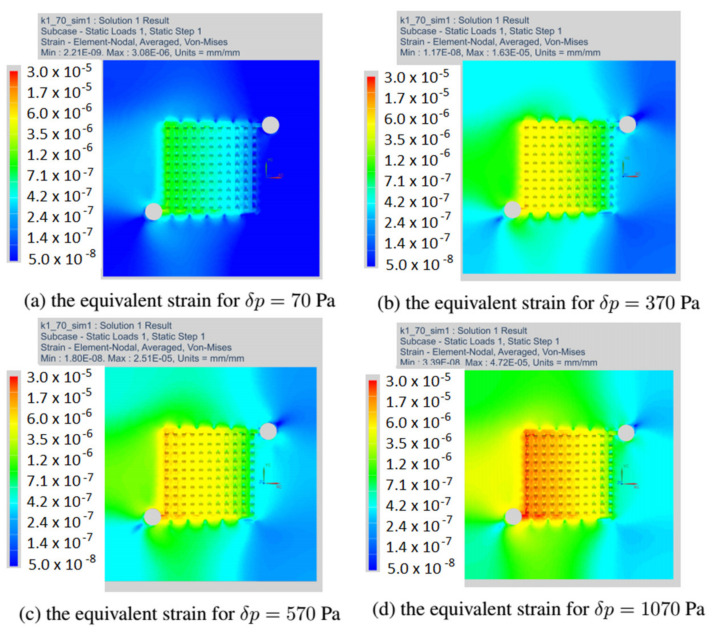
The field of equivalent strain read in the anode layer at different loads for fuel cell in variant k = 1.

Figure 14 shows the stress fields read for the variant k = 1, which had the highest current density. We have presented strain maps for the same operating points as in the case of the k = 0 variant. Although the shape change caused an increase in the current density, the strain equivalent remained the same. 

**Figure 15:**
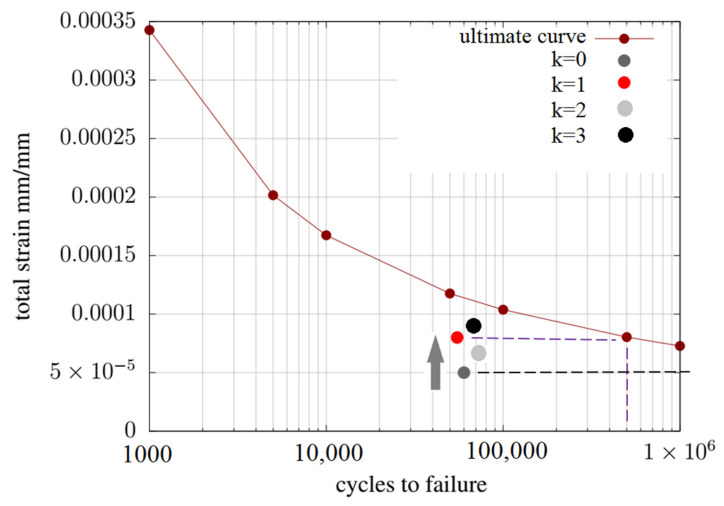
Number of cycles to fatigue estimated with Coffin–Manson procedure.

The following figure, Figure 15, shows the number of cycles to failure estimated for the variant k = 0 and generating the highest current flux, marked as k = 1. The Coffin–Manson procedure we used in the simulation and the results showed that the change in the number of cycles to failure after shape change did not change drastically. Both values are well above 100,000 cycles in the graph, which is usually the design number. 

### 3.5. Field of Equivalent Stress and Estimated Time of Creep Rupture Based on the Regression Analysis Technique 

This section discusses the stress fields obtained in all variants of the anode channel. The simulations were performed with the same load and assumed marginal conditions. To illustrate the stress fields in the entire operating range of the fuel cell, the variant with CSFF channels, marked k = 0, and the variant k = 1, with the highest power density, were selected. As already mentioned, the simulations were carried out for several loads corresponding to the operating range of the fuel cell. 

**Figure 16:**
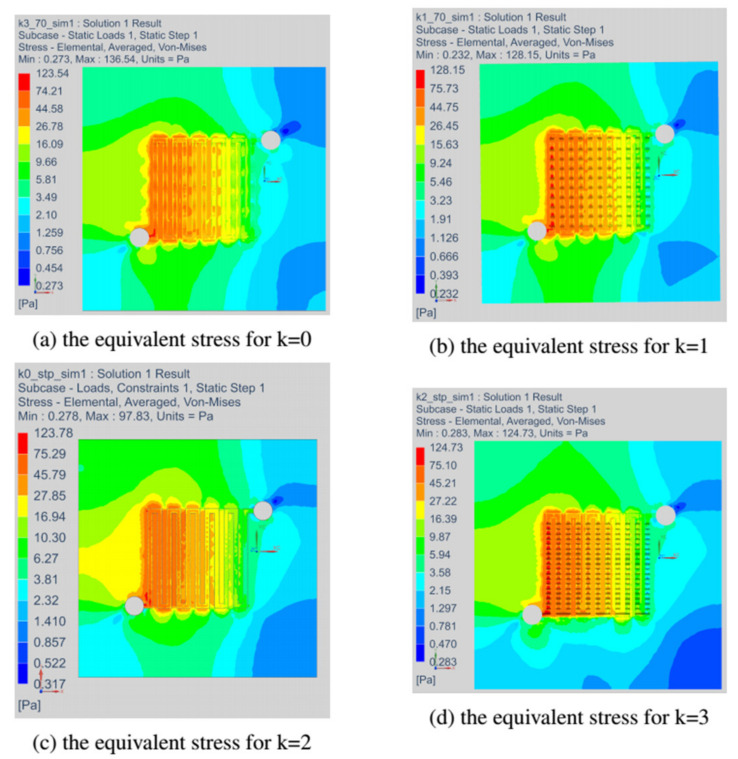
The field of equivalent stresses read at a load of 70 Pa for all variants of the anode channel.

Figure 16 shows the fields of reduced stresses read for all considered variants of the fuel cell. As already mentioned, the simulations we made with identical operating parameters of the fuel cell. For all the changes in the shape of the anode channel, the stress maps do not differ significantly, and the places with the highest material stress include the first inlet channels. 

**Figure 17:**
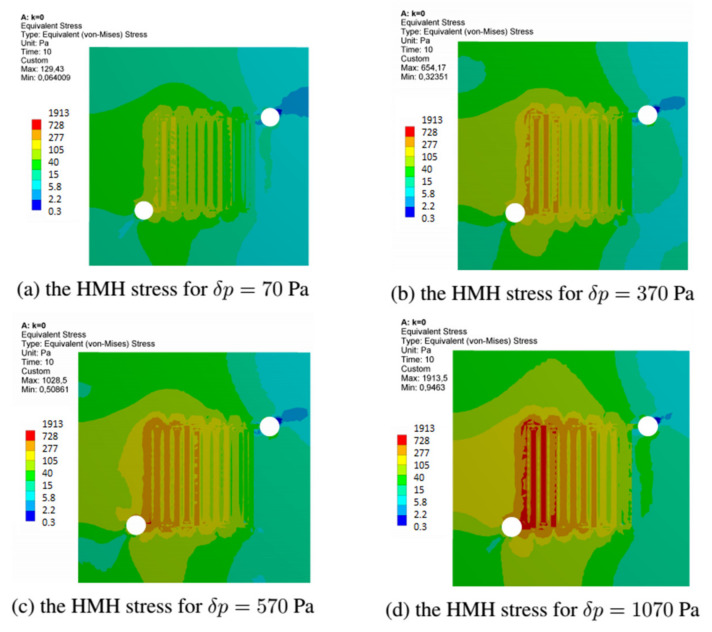
The equivalent stress field generated in the anode layer at different loads for fuel cell in variant k = 0.

In the next drawing, Figure 17, the distribution of equivalent stress fields for the variant k = 0 read at selected operating points of the fuel cell is shown. For each variant, we located the fields of maximum stresses at the beginning of the channel. The presented variant k = 0 corresponded to the CSFF channel and has the lowest current density among the considered variants of the cell. 

**Figure 18:**
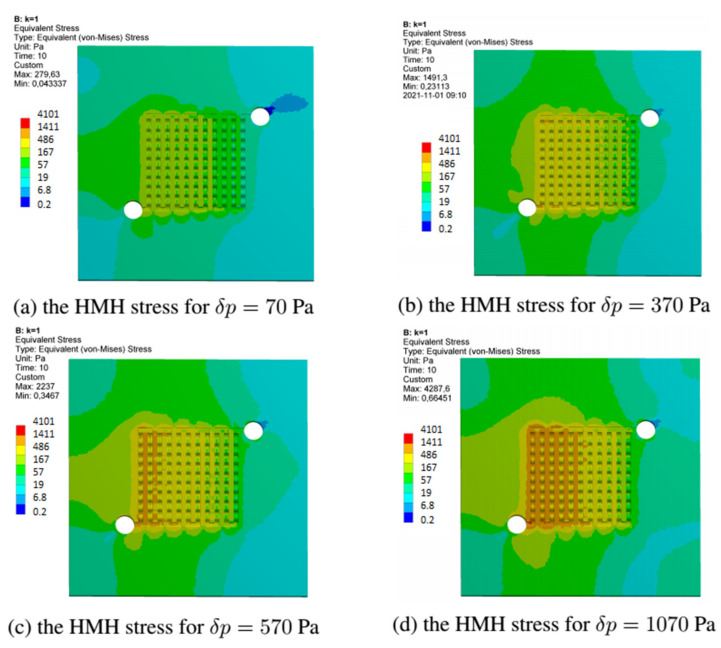
The equivalent stress field generated in the anode layer at different loads for fuel cell in variant k = 1.

Figure 18 shows the stress fields read for the variant k = 1, which had the highest current density. We have presented stress maps for the same operating points as in the case of the k = 0 variant. Although the shape change caused an increase in the current density, the stresses’ magnitude remained the same. 

**Figure 19:**
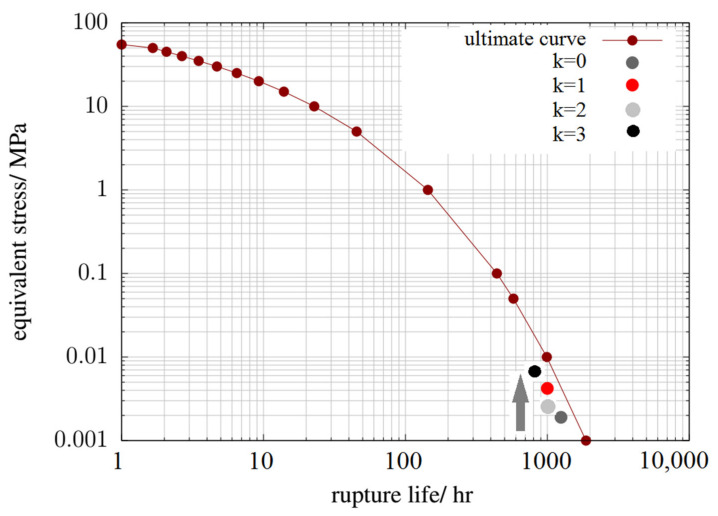
Graph of the curve: equivalent stress–time to failure due to high-temperature creep, prepared for selected variants of the anode channel.

Figure 19 shows a graph of the curve describing the value of allowable stresses as a function of the time needed for failure due to high-temperature creep. The points indicate the time to failure for the two extreme variants of the anode channel; k = 0 and k = 1. The first was a reference channel with CSFF shapes, while the second had WSFF shapes with geometry allowing for the highest current density. 

The results for the remaining variants were between the marked points. The applied geometry obtained approximately 38% higher current density when the rupture life range did not decrease significantly.

Figure 19 shows a graph of the curve describing the value of allowable stresses as a function of the time needed for failure due to high-temperature creep. The points indicate the time to failure for the two extreme variants of the anode channel; k = 0 and k = 1. The first was a reference channel with CSFF shapes, while the second had WSFF shapes with geometry allowing for the highest current density. The results for the remaining variants were between the marked points. The applied geometry obtained approximately 38% higher current density when the rupture life range did not decrease significantly.

## 4. Conclusions

In this paper, we have analyzed various variants of the anode channel shape. We analyzed several variants of the sinusoidal waves of the upper surface of the channel, and the obtained results were related to the characteristics obtained for the CSFF channel. We have shown the influence of changing the shape of the upper surface of the channel on the current–voltage characteristic curves. We also discussed how the introduced shape changes affect the image of the stress and strain fields in the anode collector. Further, we analyzed how these changes affect the number of cycles on fatigue and high temperature creep durability. The conclusions are drawn we have presented below:

- The introduction of the sinusoidal waves in the upper surface of the anode channel improved the current–voltage characteristics of the cell. The reason for this phenomenon, according to the authors, is the generation of an additional convective flux, increasing the size of the H_2_ transported to the interior of the porous medium;
- The introduction of changes in the shape of the upper surface of the channel did not cause any significant changes in the field of equivalent stresses and deformations that could be read in the anode collector;
- The introduced changes in the shape did not significantly change the number of cycles needed for the destruction;
- The introduced shape changes did not significantly change the safe operation time due to high-temperature creep.

## Figures and Tables

**Table 1:** Material properties and physical parameters for PEMFC fuel cell.

Name	Unit	Symbol	Value
Diffusion layer thickness	m	δ_GDL_	0.4 × 10^−3^
Catalyst layer thickness	m	δ_CL_	0.032 × 10^−3^
Membrane thickness	m	δ_MEM_	0.1 × 10^−3^
Channel width, height, length	mm	W_ch_, H_ch_, L_ch_	1.0, 1.0, 50
Porosity of GDL, Catalyst		ε	0.40
Membrane porosity		ε	0.28
Catalyst porosity		ε	0.40
Diffusion layer permeability	m^2^	α	1.76 × 10^−11^
Catalyst layer permeability	m^2^	α	1.76 × 10^−11^
Membrane layer permeability	m^2^	α	1.80 × 10^−18^
Electronic conductivity of GDL		κ_GDL_	Anisotropy
Electronic conductivity of CL	S m^−1^	κ_CL_	5000
Ionic conductivity of Membrane	S m^−1^	Κ_mem_	$\left(0.5139\lambda -0.326\right)$ $\exp\left(\frac{1268}{303.15}-\frac{1268}{T}\right)$
Thermal conductivity of CL, Mem, GDL	W m^−1^ K^−1^	λ_CL, MEM_,	1.0, 0.95, 20.0
Faradays Constance	C mol^−1^	F	96.485

**Table 2:** Material properties for anode/cathode collector.

Name	Unit	Symbol	Value
Young modulus at direction	GPa	E_11_	142
Young modulus at normal to direction	GPa	E_22_	13.79
Poisson’s ratio		υ_12_	0.3
Shear modulus	GPa	G_12_	4.64
Shear modulus	GPa	G_13_	4.64
Shear modulus	GPa	G_23_	3.03
Density	kg m^−3^	ρ	1.61 × 10^3^
Tensile strength in the direction	MPa	X^T^	1447
Compressive strength in the direction	MPa	X^C^	1447
Tensile strength in the normal to direction	MPa	Y^T^	51.7
Compressive strength in the normal to direction	MPa	Y^C^	206
Longitudinal shear strength	MPa	S^L^	93
Transverse shear strength	MPa	S^T^	103

**Table 3:** Detailed parameters of sinusoidal wave for case studies.

Case No.	α/mm	β/mm	γ/mm
k = 0	0	0	1
k = 1	2	0.5	1
k = 2	1	0.5	1
k = 3	4	0.5	1

**Table 4:** Physical parameters used in the validation and then numerical simulation of the PEMFC fuel cell model.

Name	Unit	Symbol	Value
Anode reference pressure	Pa	*p_ref_*	0.105 × 10^6^
Cathode reference pressure	Pa	*p_ref_*	0.105 × 10^6^
Anode pressure inlet, outlet	Pa	*p*	70, 10
Cathode pressure inlet, outlet	Pa	*p*	70, 10
Anode relative humidity		RH_A_	0.9
Cathode relative humidity		RH_C_	0.8
Reference current density	A cm^−2^	I_ref_	1.0
Reference H_2_/O_2_/H_2_O mass fraction on the inlet anode channel		cH_2_,O_2_/H_2_O	1.0, 0, 0
Reference H_2_/O_2_/H_2_O mass fraction on the inlet cathode channel		cH_2_,O_2_/H_2_O	0, 1.0, 0
Open circuit cell voltage	V	*φ*	1.17
